# Humoral Responses Elicited by Adenovirus Displaying Epitopes Are Induced Independently of the Infection Process and Shaped by the Toll-Like Receptor/MyD88 Pathway

**DOI:** 10.3389/fimmu.2018.00124

**Published:** 2018-02-05

**Authors:** Aleksandra Anchim, Najat Raddi, Lena Zig, Patrick Perrieau, Ronan Le Goffic, Bernhard Ryffel, Karim Benihoud

**Affiliations:** ^1^Vectorologie et thérapeutiques anticancéreuses, UMR 8203, CNRS, Univ. Paris-Sud, Gustave Roussy, Université Paris-Saclay, Villejuif, France; ^2^VIM, INRA, Université Paris-Saclay, Jouy-en-Josas, France; ^3^Experimental and Molecular Immunology and Neurogenetics, UMR 7355, CNRS-University of Orléans, Orléans, France; ^4^Institute of Infectious Diseases and Molecular Medicine (IDM), Cape Town, South Africa

**Keywords:** adenovirus, fiber, innate immunity, antibody isotype, MyD88, mitochondrial antiviral-signaling

## Abstract

The use of serotype 5 adenovirus (Ad)-derived vectors in vaccination is confronted to preexisting anti-Ad immunity. Epitope display on Ad capsid is currently being investigated as an alternative approach of vaccination. The present study seeks to better understand virus- and host-related factors controlling the efficacy of this new vaccination approach. In contrast to an Ad vector expressing ovalbumin as a transgene, Ad displaying an ovalbumin-derived B-cell epitope inserted into the fiber protein was able to elicit antibody responses in both Ad-naive and Ad-immune mice. Moreover, introduction of a set of mutations abrogating Ad interaction with its receptors did not modify the virus capacity to elicit a humoral response against the inserted epitope while reducing its capacity to mount antibody responses against the transgene product. Taken as a whole these data indicate that the efficacy of Ad displaying epitopes requires neither Ad binding to its receptors nor the infection process. In addition, the use of genetically deficient mice demonstrated that both toll-like receptor (TLR)/MyD88 and RIG-I/mitochondrial antiviral-signaling (MAVS) innate immunity pathways were dispensable to mount anti-epitope antibody responses. However, they also revealed that TLR/MyD88 pathway but not RIG-I/MAVS pathway controls the nature of antibodies directed against the displayed epitope.

## Introduction

Adenoviruses (Ad) belong to a family of non-enveloped DNA viruses containing a linear double-strand DNA genome. Knowledge accumulated over more than 20 years on their biology has led to the development of Ad-derived vectors (1). Ease of Ad manipulation, their production at high titers, as well as the strong level of gene expression achieved by these vectors makes them an attractive tool not only for gene therapy but also for vaccination. Indeed, Ad-mediated gene transfer of DNA fragments encoding heterologous proteins was shown to elicit strong humoral and cellular responses toward transgene-encoded proteins (2). The efficacy of this approach of vaccination (hereafter referred to as the classical approach) stems from Ad’s ability to transduce *in vivo* a large set of cells and in the intrinsic immunogenic properties of this vector (3).

Several studies investigated Ad capsid proteins and cell receptors controlling Ad infection. Thus, in the case of the well-characterized serotype 5 Ad (Ad5), interaction of fiber protein, and more precisely its knob, with Coxsackie and Ad receptor (CAR) was shown to be responsible for initial virus attachment. Subsequent binding of penton base-located RGD motif to cellular integrins allows virus endocytosis through a clathrin-dependent pathway (3). The role of integrins and CAR in controlling Ad distribution *in vivo* was, for a long time, a matter of debate. CAR was shown to play a minor role in the transduction of different tissues, including liver and spleen (4, 5). Integrin-ablated Ad led to a reduced transgene expression in spleen and lungs (6). Of note, ablation of both CAR and integrin binding was unable to reduce liver gene transfer (5, 7) [for review, see Ref. (3)]. Besides CAR and integrins, different studies demonstrated a role for of Ad shaft in controlling liver and spleen transduction (4, 8, 9). More recently, different Ad serotypes including serotype 5 were shown to bind to plasma proteins such as vitamin K-dependent coagulation factors, leading to liver transduction (10). Among numerous coagulation factors, factor X (FX) plays a key role in liver transduction by bridging Ad capsid to liver heparan sulfate proteoglycans. Moreover, mutations of Ad capsid helped to identify Ad hexon protein as the capsomer directly involved in FX binding (11–13).

Apart from their role in cell transduction, Ad receptors contribute to the intrinsic immunogenic properties of this vector. For example, interaction with CAR and integrins were at the origin of pro-inflammatory cytokine and chemokine production in epithelial cells and macrophages [for review, see Ref. (3)]. Innate immune responses to Ad are also triggered through the stimulation of pathogen recognition receptors. Several studies reported a role of membrane-anchored sensors, such as toll-like receptor (TLR) 9 and more surprisingly TLR2 in controlling cytokine production (14, 15). In addition, mice deficient in Myeloid differentiation primary response gene 88 (MyD88)—an adaptor protein common to different TLR signaling pathways—displayed reduced levels of plasma pro-inflammatory cytokines and chemokines upon intravenous Ad administration (14). After endosome escape, one could anticipate Ad to stimulate cytosolic sensors. Indeed, following Ad infection, synthesis of viral-associated RNA elicits type I interferon (IFN) through retinoic acid-inducible gene (RIG)-I mediated pathway (16). Finally, comparison of the transcriptome in the spleen after administration of wild-type and FX-ablated Ad revealed an unanticipated key role of FX in activating NFκB pathway leading to pro-inflammatory cytokine production (17).

Despite their efficacy in transducing cells *in vivo* and their strong adjuvant properties, the use of Ad in the classical vaccination approach is hampered by the highly prevalent anti-Ad5 immunity. Moreover, Ad vector immunogenicity impairs the efficiency of homologous prime-boost administrations. Several strategies were developed to overcome these limitations [for review, see Ref. (2)]; among them, epitope display relying on genetic insertion of relevant epitopes on Ad capsid. This approach was successful at inducing antibody responses against *P. aeruginosa* (18), *B. anthracis* (19), or *Plasmodium* (20). Using a B cell epitope derived from a model antigen, ovalbumin, we previously uncovered that anti-Ad preexisting antibodies (Abs) strongly increased the antibody response elicited by Ad displaying the epitope into the fiber protein (21). The present results seek to go further in our understanding of this strategy of vaccination by defining the role of Ad interaction with their receptors, as well as the influence of innate immune pathways.

## Materials and Methods

### Mice

Seven-week-old C57BL/6 female mice were purchased from Harlan (Gannat, France). MyD88- (MyD88^−/−^) (22) and mitochondrial antiviral-signaling (MAVS)- (MAVS^−/−^) (23) deficient mice were bred in animal facilities of TAAM-UPS 44 (Orléans) and UMR 0892 (VIM, Jouy-en-Josas), respectively. All mice were conditioned for at least 1 week in our animal facilities before beginning of the experiments. All animal experiments were approved by Ethics Committee No. 26 (officially recognized by the French Ministry for Research) in accordance with the European Directive 2010/63 UE and its transposition into French Law.

### Virus Construction and Production

AdWT [described as AE18 in Ref. (24)] is based on Ad5 and is deleted in E1 and E3 regions. The expression cassette cloned instead of the E1 region contains the promoter/enhancer from the immediate early gene of human cytomegalovirus, the *Escherichia coli lacZ* gene with a nuclear localization signal, and the SV40 late polyA signal (SV40pA). AdH-3OVA2 and AdF-3OVA2 derived from AdWT and displaying OVA_320–341_ (3OVA2) epitope, respectively, in hexon or fiber protein were described previously (21). AdP*F-3OVA2, AdH*F-3OVA2, AdH*P*F-3OVA2, and AdS*F-3OVA2 disabled to a different extend in Ad interactions with their natural receptors, were derived from AdF-3OVA2 (Table 1). More precisely, AdP*F-3OVA2 was derived from AE74 (9) bearing a deletion of penton base RGD motif, impairing interaction with integrins. AdH*F-3OVA2 was derived from AdH[GA]24 (12) bearing a deletion of hypervariable region 5 (HVR5) of hexon protein, impairing FX binding. AdH*P*F-3OVA2 contains both deletions. Finally, AdS*F-3OVA2 was obtained from AdWT by replacing the fiber shaft with the shaft from Ad serotype 3 (25). All capsid-modified viruses (Table 1) were constructed by recombinational cloning in *E. coli* (26).

**Table 1:** Characteristics of Ad displaying ovalbumin-derived epitopes.

Virus	Transgene	Capsid modifications			Titer^b^ (×10^12^ vp/cell)
		Penton	Hexon	Fiber	
AdWT	βgal	–	–	–	4.4 ± 0.8
AdOVA	Ovalbumin	–	–	–	7.3 ± 2.1
AdH-3OVA2	βgal	–	3OVA2^a^	–	4.5 ± 2.9
AdF-3OVA2	βgal	–	–	3OVA2	4.6 ± 1.9
AdP*F-3OVA2	βgal	RGD deletion	–	3OVA2	2.3 ± 1.0
AdH*F-3OVA2	βgal	–	Hypervariable region 5 (HVR5) deletion	3OVA2	2.1 ± 0.6
AdH*P*F-3OVA2	βgal	RGD deletion	HVR5 deletion	3OVA2	2.5 ± 1.2
AdS*F-3OVA2	βgal	–	–	3OVA2 + Ad3 shaft	1.7 ± 0.6

*^a^3OVA2 refers to ovalbumin 320–341 epitope*.

*^b^Mean ± SD*.

AdOVA (provided by Dr. D. Descamps, INRA, Jouy-en-Josas) has a wild-type capsid and encodes the complete amino-acid sequence of ovalbumin protein. AdControl encoding no transgene was described previously (27).

All viruses were obtained using previously described procedures (5), stored at −80°C in PBS-7% glycerol, and titrated by spectrophotometry [1 OD_260_ = 1.1 × 10^12^ viral particle (vp)/ml].

### Cell Lines

The 293A human embryonic kidney cell line (R705-07, Invitrogen) was maintained in modified Eagle medium supplemented with 10% FBS and 1% of non-essential amino acids. CHO-k1-hCAR and CHO-k1-pCDNA were kindly provided by J. M. Bergelson (School of Medecine, University of Pennsylvania). L929 murine fibrosarcoma cells were kindly provided by Dr. U. Greber (Institute of Molecular Biology, Zurich, Switzerland) and maintained in DMEM supplemented with 10% FCS.

### SDS-PAGE and Western Blot

Purified viruses (10^10^ vp) were resuspended in Laemmli lysis buffer, boiled for 10 min and loaded onto a 10% NuPage gel (Novex, Invitrogen, CA, USA). After electrophoresis, the gel was stained with a silver staining kit (Invitrogen, Carlsbad, CA, USA). Alternatively, the gel was transferred on nitrocellulose membrane and the membrane was incubated with a rabbit polyclonal antibody directed against the fiber protein.

### *In Vitro* Cell Transduction

In order to evaluate virus infectivity, cells were plated out in 12-well plates at 1 × 10^5^ cells/well 48 h prior to infection. On the day of infection, cells were counted and infected with the indicated multiplicity of infection (MOI) of different Ad in 400 µl of serum-free medium. After 24 h, βgalactosidase (βgal) activity was measured using a chemiluminescent assay (BD Biosciences, Clontech, CA, USA) and protein concentration was determined using the Bio-Rad Protein Assay (Bio-Rad Laboratories, Hercules, Marnes-la-Coquette, France). Results were presented as relative light units (RLU) per microgram of proteins.

In order to evaluate FX-dependent cell transduction, viruses were mixed with or without human FX (1 U/ml, Cryopep). Then, virus-FX solution was added to CHO-k1-pCDNA cells and cells were incubated for 24 h at 37°C. βgal activity was measured as described above.

### Epitope Detection on Virions

To confirm the presence and accessibility of the epitopes on the capsid surface, viral particles were coated on 96-well plates (Nunc, Roskilde, Denmark). Viruses were inactivated by incubation at 56°C for 30 min, followed by addition of 0.1% SDS. Spectrophotometric measurements at 215 and 225 nm allowed to determine viral protein concentrations, and subsequently 100 ng was coated on 96-well plates. To analyze epitope presence and accessibility on virions, non-denaturated viruses (100 ng) were coated on the plates. After overnight incubation at 4°C, non-specific sites were blocked with 5% milk PBS-Tween for 2 h, then plates were washed and incubated for 1 h with an anti-ovalbumin rabbit polyclonal antibody (AB1225, Millipore, MA, USA). Upon washing, an anti-rabbit IgG peroxidase-linked Ab (NA934, Amersham Biosciences, Saclay, France) was added for 1 h and peroxidase activity was revealed by incubation with the substrate *O*-phenylenediamine dihydrochloride (Sigma-Aldrich, Lyon, France) for 30 min. The reaction was stopped by addition of 3 N HCl and spectrophotometric readings were performed at 490 nm. Each virus was assayed in sexdecaplicates and the experiments were repeated at least twice.

### *In Vivo* Experiments

Capsid-modified viruses (10^10^ vp) in PBS (200 µl) were injected intraperitoneally. Repeated injections were performed at 2 weeks intervals with a total number of injections ranging between two and three. Blood samples were collected from the submandibular vein, before virus injection and at different time points thereafter. Mice sera were prepared and analyzed for the presence of anti-ovalbumin, anti-βgal, and anti-Ad Abs by ELISA as described below.

In some experiments, mice were depleted of coagulation factors by subcutaneous injection of 133 µg of warfarin in 100 µl of PBS at days 3 and 1 prior to virus administration as described previously (10). Ad-naive or Ad–immune mice were obtained after injection of PBS or AdControl (10^10^ vp), respectively.

### Measurement of Specific Abs

Sera were analyzed for the presence of specific Abs by ELISA. After coating of 96-well plates (Nunc) with 1 µg of ovalbumin (Sigma), 100 ng of βgal (Sigma), or 100 ng of denaturated AdWT viral particles, serial dilutions of the sera in 5% milk PBS-Tween were added. Bound Abs were detected with peroxidase-conjugated anti-mouse IgG, IgG1, IgG2b, or IgG2c isotype goat Abs (Southern Biotechnology Associates, Birmingham, AL, USA). The peroxidase activity was revealed by incubation with the substrate *O*-phenylenediamine dihydrochloride (Sigma-Aldrich) for 30 min. The reaction was stopped by addition of 3 N HCl and spectrophotometric readings were performed at 490 nm. Titers were defined as the reciprocal of the highest dilution giving an OD_490_ twofold above background values.

### Statistical Analysis

Data from *in vivo* experiments (titers) were log_2_-transformed before analysis. Comparison between two groups was done using unpaired Mann–Whitney test. Comparison between multiple groups were done using Kruskal–Wallis test followed with Dunn’s *post hoc* test. Two-way repeated measures ANOVA was used for comparison of responses measured for different groups at different time points, then Bonferroni *post hoc* test was used to compare between groups at each time point. Differences were considered significant when *P* < *0.05*. All graphs and statistical tests were obtained with the use of GraphPad Prism software.

## Results

### Antibody Responses Elicited by Ad Displaying Epitopes Do Not Require Gene Transfer

**Figure 1:**
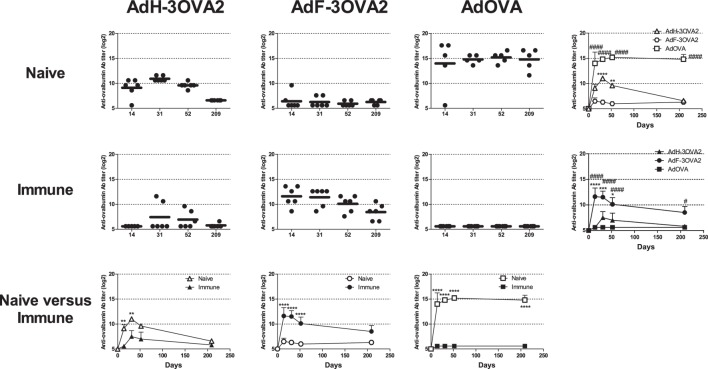
Kinetics of anti-ovalbumin humoral response. Naive- (upper panel) or Ad-immune (medium panel) C57Bl/6 mice were immunized intraperitoneally with 10^10^ vp of capsid-modified Ad (AdH-3OVA2 or AdF-3OVA2) or with an Ad vector encoding ovalbumin as a transgene (AdOVA). Anti-ovalbumin IgG titers were determined by ELISA at different time points after injection. Titers below 100 were plotted as 50. Left part, circles and bars represent results of individual mice (*n* = 5–6) and means, respectively. Right part, means + SEM for each group. *p < 0.05, **p < 0.01, ***p < 0.001, ****p < 0.0001 difference between AdF-3OAV2 and AdH-3OVA2. ^#^*p* < 0.05, ^###^*p* < 0.001, and ^####^*p* < 0.0001 difference between AdF-3OVA2 and AdOVA. (Lower panel) comparison between naive and immune mice. **p < 0.01 and ****p < 0.0001.

Our previous results have shown that pre-existence of anti-Ad Abs strongly enhances anti-ovalbumin antibody responses elicited by AdF-3OVA2 bearing OVA_320–341_ B cell epitope (21), suggesting that cell transduction is dispensable for the efficacy of this vector. To address specifically this point, we compared the ability of capsid-modified AdF-3OVA2 and AdH-3OVA2 (Table 1) to elicit anti-ovalbumin antibody responses relative to AdOVA, encoding the whole ovalbumin protein as a transgene, in both naive and immune mice. C57BL/6 mice were injected with PBS or with an Ad bearing a wild-type capsid (AdWT, 10^10^ vp) and 2 weeks later received one intraperitoneal injection of AdH-3OVA2, AdF-3OVA2, or AdOVA (10^10^ vp). In Ad-naive mice, AdOVA induced high levels of anti-ovalbumin Abs from day 14 p.i. up to 209 days p.i (Figure 1, upper panel). In accordance with our previous study (21), AdH-3OVA2 led to a significantly higher anti-ovalbumin Ab responses compared to AdF-3OVA2. However, these responses remain lower than the ones observed in AdOVA-injected mice (Figure 1, upper panel). In Ad-immune mice, AdF-3OVA2 triggered strong anti-ovalbumin antibody responses compared to AdH-3OVA2 as described previously (21) while AdOVA was unable to trigger any antibody response (Figure 1, middle panel). Interestingly, the immune responses elicited by AdF-3OVA2 were long-lasting with strong Ab titers detectable up to 7 months after the immunization. While AdF-3OVA2 and AdH-3OVA2 elicit strong anti-βgalactosidase (βgal) Ab responses in Ad-naive mice, none of them triggered significant Ab responses in Ad-immune mice (Figure S1 in Supplementary Material).

Altogether, our data indicate that, in contrast to AdOVA, AdF-3OVA2 was able to trigger Ab responses in both Ad-naive and Ad-immune mice, suggesting that virus transduction is not mandatory for the efficacy of this vector.

### Production and Characterization of Ad Displaying Ovalbumin Epitope in the Fiber Protein and Ablated in their Native Receptor Interactions

**Figure 2:**
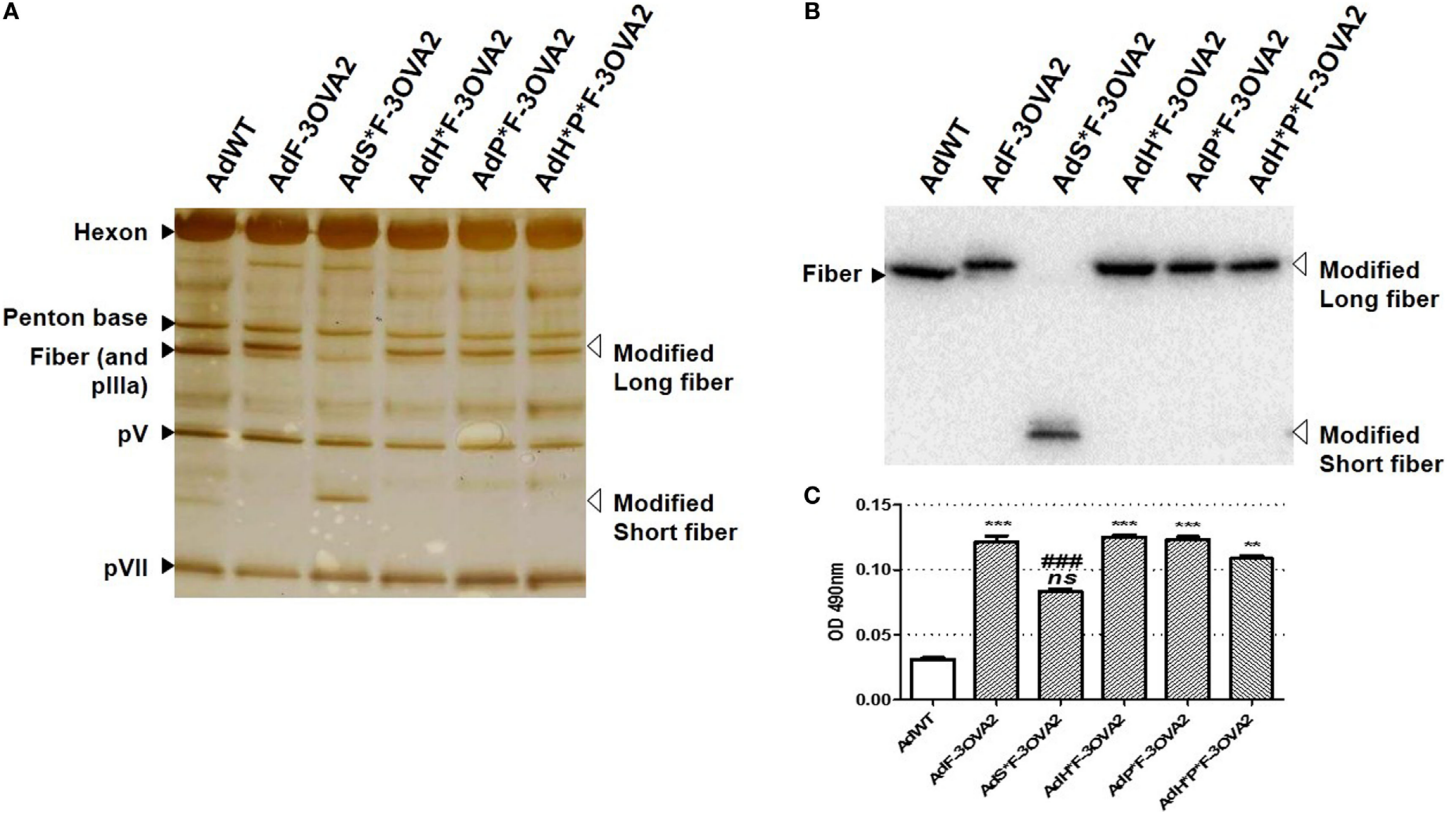
Epitope detection on capsid-modified vectors. **(A)** Silver staining of capsid-modified Ad. Similar amount (10^10^ vp) of either a control Ad (AdWT) or a capsid-modified Ad (AdF-3OVA2, AdH*F-3OVA2, AdP*F-3OVA2, AdH*P*F-3OVA2, and AdS*F-3OVA2) were separated on a 10% polyacrylamide gel. Modified fibers are indicated with white arrows while hexon, penton base, polypeptide IIIa (pIIIa), and native fiber proteins are labeled with black arrows. **(B)** Detection of fiber protein by western blot. **(C)** Detection of 3OVA2 epitopes on virions. ELISA plates were coated with 100 ng of native viruses and incubated with a rabbit polyclonal antibody against ovalbumin protein. The binding was detected with a HRP-conjugated secondary antibody. One of two experiments is shown, means + SEM of 16 replicates. ns, non significant; **p < 0.01 and ***p < 0.001 versus AdWT; ^###^*p* < 0.001 versus AdF-3OVA2.

To examine more precisely whether virus transduction plays any role in controlling the efficacy of AdF-3OVA2, different vectors were produced displaying both 3OVA2 epitope inserted into the fiber protein and capsid modifications impairing binding to specific receptors. AdP*F-3OVA2 presents a deletion of the RGD motif in penton base protein in order to ablate interaction with integrins (9). AdH*F3-OVA2 contains a mutation in hexon HVR5 impairing binding to FX (12). AdH*P*F-3OVA2 possesses both hexon and penton base mutations. Finally, AdS*F-3OVA2 contains an Ad3 fiber shaft instead of Ad5 shaft (25) impairing binding to CAR receptor. All vectors were produced at titers comparable to AdF-3OVA2 (Table 1). SDS-PAGE analyses showed no difference in virus composition between the different Ad. As expected, all viruses but AdS*F-3OVA2 displayed a similar migration pattern for the modified fiber (MW = 63.3 kDa, Figure 2A). For AdS*F-3OVA2, the migration pattern of the fiber protein is consistent with its reduced size (MW = 33.8 kDa, Figure 2A). Using a polyclonal serum specific for the fiber protein, we confirmed the modification of fiber size (long fiber or short fiber) for all vectors displaying 3OVA2 epitope (Figure 2B). Additionally, 3OVA2 epitope was detected on native virions by ELISA using a polyclonal anti-ovalbumin antibody (Figure 2C). Interestingly, the detection of 3OVA2 epitope on AdS*F-3OVA2 was reduced compared to AdF-3OVA2 (*p* < 0.001).

**Figure 3:**
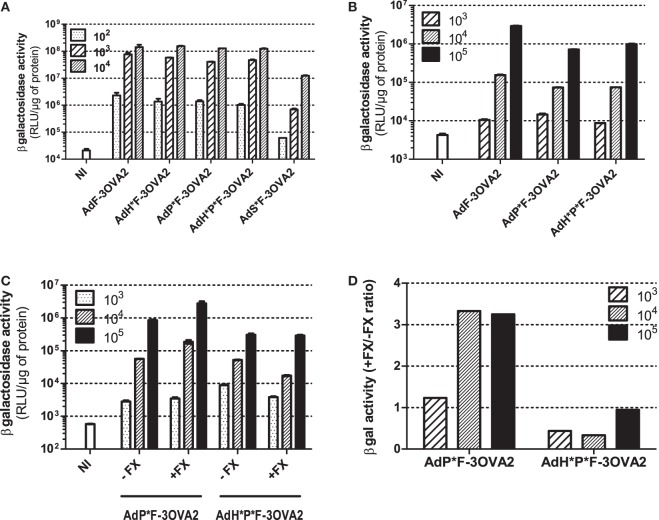
*In vitro* infectivity of capsid-modified vectors. **(A)** Transduction of CHO-k1-hCAR. Cells were mock-infected (NI) or infected with indicated multiplicity of infection (MOI) of capsid-modified Ad (AdF-3OVA2, AdH*F-3OVA2, AdP*F-3OVA2, AdH*P*F-3OVA2 and AdS*F-3OVA2). Vectors were added at different MOI to CHO-k1-hCAR cells and incubated for 24 h. **(B)** Transduction of L929 cells. Cells were mock-infected or infected with different MOI of capsid-modified Ad (AdF-3OVA2, AdP*F-3OVA2 and AdH*P*F–3OVA2). **(C,D)** Transduction of CHO-k1-pCDNA. Cells were mock-infected or infected with different MOI of capsid-modified Ad (AdH*F-3OVA2 and AdH*P*F-3OVA2) with or without physiological levels (1 U/ml) of factor X (FX). In all panels, βgal activity (means + SD of duplicates) was measured in cells harvested 24 h post-infection and expressed as relative light unit (RLU) per microgram of protein **(A–C)** or as a ratio of βgal activity with FX relative to the one without FX **(D)**. The representative results of at least two experiments are shown.

With the exception of AdS*F-3OVA2, all vectors possess the same capacity to transduce CHO-k1-CAR cells, a cell line overexpressing Ad primary receptor. AdS*F-3OVA2 led to a reduced cell transduction (Figure 3A) consistent with previous results of our laboratory (Raddi et al., in revision). Next, we examined transduction efficiency using specific cell lines allowing to monitor the detargeting from native Ad receptors. AdP*F-3OVA2 and AdH*P*F-3OVA2 were tested on CAR-negative integrin-positive L929 cells. Both vectors led to a reduced βgal activity compared to AdF-3OVA2, confirming their reduced ability to bind integrins (Figure 3B). AdP*F-3OVA2 and AdH*P*F-3OVA2 were used to transduce CAR-negative CHO-pcDNA cells in the presence or absence of FX (Figure 3C). The results showed that both vectors displayed reduced ability to use FX in accordance with the role of hexon protein in FX binding (Figures 3C,D).

### Humoral Responses Elicited by Ad Displaying Ovalbumin Epitope on the Fiber Protein and Ablated in their Native Receptor Interactions

**Figure 4:**
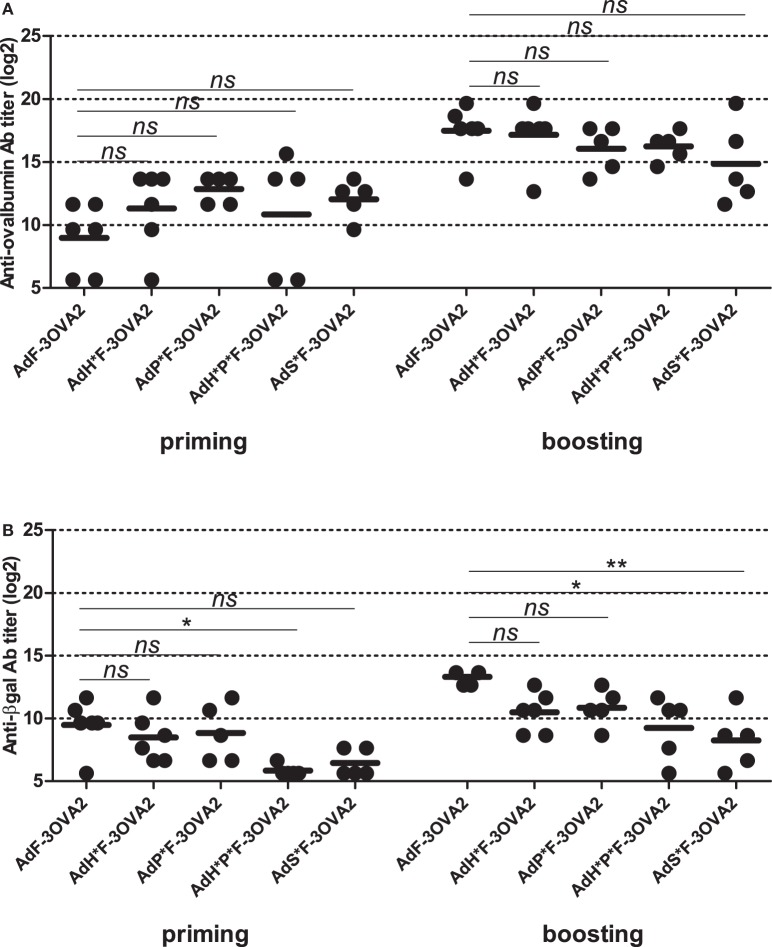
Humoral responses elicited by Ad displaying 3OVA2 epitopes and ablated in their native receptor interaction. C57BL/6 mice were immunized intraperitoneally with 10^10^ vp of capsid-modified AdF-3OVA2, AdH*F-3OVA2, AdP*F-3OVA2, AdH*P*F-3OVA2, and AdS*F-3OVA2. Anti-ovalbumin **(A)** and anti-βgal **(B)** IgG titers were determined by ELISA at day 14 after first (priming) and second (boosting) administration. Titers below 100 were plotted as 50. One of two experiments is shown, dots and bars represent results from individual mice (*n* = 5–6 mice) and means, respectively (*p < 0.05; **p < 0.01 versus AdF-3OVA2).

After the characterization of produced vectors, we examined their capacity to mount anti-ovalbumin humoral responses. C57BL/6 mice were injected intraperitoneally twice 2 weeks apart with 10^10^ vp. Sera were collected 2 weeks after each administration and anti-ovalbumin, anti-βgal, and anti-Ad Abs were quantified by ELISA. Remarkably, no difference in ability to trigger anti-ovalbumin Abs was observed between detargeted vectors (AdH*F-3OVA2, AdP*F-3OVA2, AdH*P*F-3OVA2, and AdS*F-3OVA2) and AdF-3OVA2, neither after priming nor after boosting (Figure 4A). In contrast, anti-βgal antibody titers were significantly reduced after priming in AdH*P*F-3OVA2-injected mice (Figure 4B, *p* < *0.05*) compared to AdF-3OVA2-injected mice. After boosting AdH*P*F-3OVA2- and AdS*F-3OVA2 were significantly impaired in their ability to trigger anti-βgal Ab (Figure 4B, *p* < 0.05 and *p* < 0.01 respectively). Of note, all vectors induced comparable anti-Ad Ab responses at all time points (data not shown). Altogether, these results underline that while detargeting Ad from its natural receptors reduces its ability to trigger humoral responses against the transgene, it has no impact on its ability to trigger humoral responses against an epitope displayed on the capsid.

In parallel to FX-detargeting through hexon modification, we also used a pharmacological approach based on administration of warfarin, a drug able to deplete all vitamin K-dependent blood factors. To do so, mice were pre-treated with warfarin or PBS before each AdF-3OVA2 administration. Blood factor depletion was confirmed by the measurement of FX activity in mice sera harvested prior to virus delivery (data not shown). Measurement of anti-ovalbumin titers showed no significant difference between warfarin- and PBS-pre-treated mice neither after priming nor after boosting (Figure S2A in Supplementary Material). Additionally, levels of anti-βgal and anti-Ad Abs also remained unmodified in warfarin-pre-treated mice (Figures S2B,C in Supplementary Material). Thus, both genetic (Figure 4A) and pharmacological (Figure 2A in Supplementary Material) approaches used to detarget Ad from FX were unable to decrease humoral responses toward 3OVA2 epitope.

Collectively, these results underline that in contrast to humoral responses against βgal transgene, humoral responses against 3OVA2 epitope displayed on the fiber protein do not rely on Ad interaction with their cellular receptors.

### Role of Innate Immune Pathways in Humoral Responses Elicited by Ad Displaying Ovalbumin Epitope Inserted into the Fiber Protein

**Figure 5:**
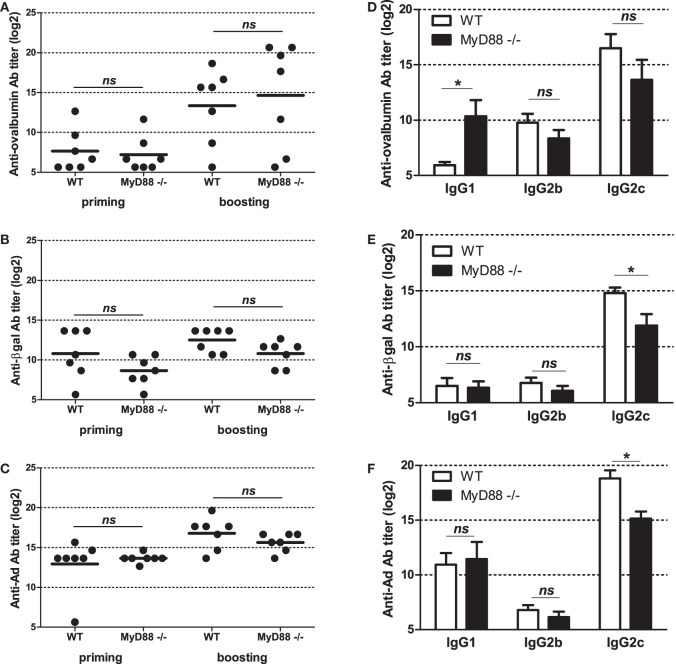
Influence of MyD88 on humoral responses elicited by Ad displaying 3OVA2 epitope. Mice were immunized intraperitoneally with 10^10^ vp of AdF-3OVA2. Anti-ovalbumin **(A)**, anti-βgal **(B)**, and anti-Ad **(C)** IgG were measured by ELISA at day 14 after first (priming) and second (boosting) administration. Dots and bars represent results from individual mice (*n* = 6–7 mice) and means, respectively. Titers below 100 were plotted as 50. ns, non significant. Anti-ovalbumin **(D)**, anti-βgal **(E)**, and anti-Ad **(F)** antibodies of IgG1, IgG2b, and IgG2c isotypes were measured by ELISA at day 42 after the first administration. One of two experiments is shown, means + SEM (*n* = 6–7). *P < 0.05.

**Figure 6:**
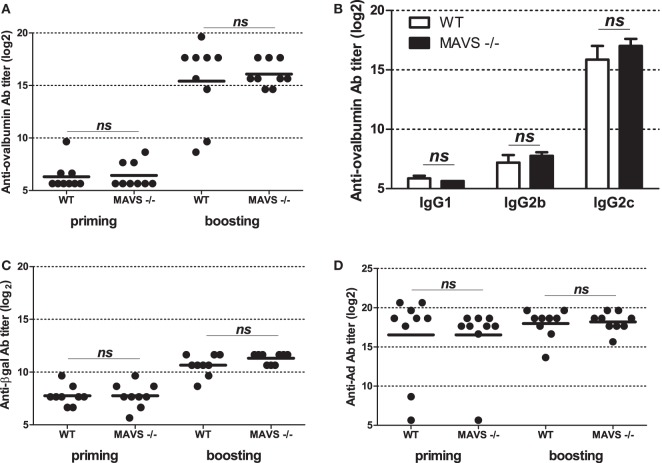
Influence of RIG-I/mitochondrial antiviral-signaling (MAVS) pathway on humoral responses elicited by Ad displaying 3OVA2 epitope. Mice were immunized intraperitoneally with 10^10^ vp of AdF-3OVA2. Total IgG specific for ovalbumin **(A)**, βgal **(C)**, and Ad **(D)** were determined by ELISA at day 14 after first (priming) and second (boosting) administration. One of two experiments is shown. Titers below 100 were plotted as 50. Circles represent individual mice (*n* = 8–9) and bars reflect means. ns, non significant. **(B)** IgG isotypes specific for ovalbumin were determined by ELISA at day 28 after the second administration. Means + SEM (*n* = 8–9). ns, non significant.

To get further insight into molecular bases controlling the efficacy of vaccination with Ad displaying epitopes, we investigated the role of innate immune pathways. First, since TLR and MyD88 were shown to participate in Ad innate immunity, we examined their role in shaping humoral responses by using MyD88-deficient mice. No significant difference was found in anti-ovalbumin IgG Ab responses in wild-type and MyD88-deficient mice, neither after priming nor after boosting (Figure 5A). In addition, both strains elicited comparable levels of anti-βgal (Figure 5B) and anti-Ad Abs (Figure 5C). Interestingly, compared to AdF3-OVA2-injected wild-type mice, AdF3-OVA2-injected MyD88^−/−^ mice displayed a strong increase in IgG1 anti-ovalbumin Abs (Figure 5D, *p* < 0.05) and a trend toward reduced levels of IgG2b and IgG2c anti-ovalbumin Abs. In contrast, MyD88^−/−^ mice displayed very low titers of anti-βgal IgG1 and IgG2b and a strong reduction in IgG2c (Figure 5E, *p* < 0.05). A reduction in IgG1 anti-Ad Abs was also found in MyD88^−/−^ mice (Figure 5F, *p* < 0.05). Then, we examined the role of RIG-I-induced innate immune pathway using mice deficient in MAVS protein. The levels of total IgG (Figure 6A) as well as IgG1, IgG2b, and IgG2c anti-ovalbumin Abs (Figure 6B) were comparable in wild-type and in MAVS^−/−^ mice, ruling out a major role of RIG-I/MAVS pathway in controlling anti-epitope humoral responses. In addition, no difference was found in anti-βgal (Figure 6C) or anti-Ad (Figure 6D) Ab responses between both strains.

Altogether, these results underline that TLR/MyD88 and RIG-I pathways are both dispensable in mounting antibody responses against 3OVA2 epitope. However, they also reveal that TLR/MyD88 pathway influences the isotype nature of antibody responses against the displayed epitope.

## Discussion

Vectors derived from Ad were used in different preclinical studies as well as in clinical trials for the vaccination purpose. The high seroprevalence of neutralizing Abs as well as the induction of strong anti-vector immunity after first administration led to the development of different strategies allowing to overcome these limits (2). Among them is the use of other Ad serotypes or even xenotypes, but also the epitope display on Ad capsid. This latter approach relies on the insertion of peptides within a capsid protein (most frequently the hexon protein). Commonly, those peptides were B cell epitopes and they were found to successfully elicit antibody responses against model antigens (21) but also against several pathogens such as influenza virus (18), *B. anthracis* (19), or *Plasmodium yoelii* (20). While several studies investigated the role of the epitope insertion site (and thus the number of introduced motifs per capsid) (21, 27) or the size of the peptides (19), to the best of our knowledge no study investigated molecular mechanisms controlling the efficacy of Ad displaying epitopes.

In a previous study, we discovered a key role of anti-Ad Abs in increasing antibody responses induced by Ad displaying ovalbumin-derived epitopes in the fiber protein. At the same time, anti-Ad Abs were impeding the efficacy of Ad displaying epitopes into the hexon protein (21). These previous results suggested that when anti-Ad Abs were able to neutralize the particle, Ad displaying epitopes were still able to trigger antibody responses. Two observations of the present paper confirmed that Ad infectious process is not mandatory to allow triggering of antibody responses by AdF-3OVA2. First, *lacZ* recombinant AdF-3OVA2 failed to mount significant anti-βgal Ab responses in Ad-immune mice while being more efficient in inducing anti-ovalbumin Ab responses than in Ad-naive mice (Figure 1). Second, detargeting AdF-3OVA2 from its native receptors reduced its ability to elicit antibody responses against βgal transgene without significant impairment of Ab production against 3OVA2 epitope (Figure 4A). Altogether, these results indicate that epitope display strategy does not rely on gene delivery in contrast to the classical Ad vaccine approach that requires transgene expression.

Detargeting Ad from integrin receptor or from FX did not impact Ad’s capacity to induce humoral responses toward βgal transgene. However, ablation of both integrin and FX binding (AdH*P*F-3OVA2) or modification of Ad5 shaft (AdS*F-3OVA2) translated into a significant reduction of anti-βgal antibody titers (Figure 4B). These results may stem from the reduced ability of AdH*P*F-3OVA2 to transduce the spleen and to induce cytokine production, due to its impaired hexon:FX and penton:integrin binding (17, 28). The decrease in anti-βgal responses for AdS*F-3OVA2 could be related to the reduced ability of shaft-mutated Ads to transduce different cells and tissues *in vivo* (Raddi et al. in revision) but may also be linked to their reduced potential to trigger pro-inflammatory cytokine and chemokine production (25).

Innate immune responses are key factors in the establishment of adaptive immune responses. Among the different innate immune pathways, Ad was shown to activate TLR/Myd88 signaling. At all analyzed time points, no significant modification of total anti-IgG Abs against Ad, βgal or 3OVA2 epitope was found in MyD88^−/−^ mice compared to their wild-type counterparts (Figure 5A). These data suggest that TLR/MyD88 pathway is dispensable in mounting efficient humoral responses or, alternatively, that other innate immune pathways could compensate the lack of MyD88. However, it should be noticed that Hartman et al. reported previously a reduction in anti-Ad IgG Ab in MyD88^−/−^ mice compared to heterozygous MyD88^+/−^ mice (29). The discrepancies between their and our study could be linked to differences in mouse strains, virus dose or mode of administration.

Increase of anti-epitope IgG1 Abs (Figure 5D) unraveled a role of MyD88 in shaping Ig isotype balance. Interestingly, MyD88 was not mandatory for the production of different anti-βgal (Figure 5E) and anti-Ad (Figure 5F) IgG isotypes, but it influences the level of production of anti-βgal and anti-Ad IgG2c production. The difference in MyD88 requirement for the production of Abs against the inserted epitope, the vector or the transgene product could be linked to intrinsic nature of the antigen (soluble protein or particle, monomeric or multimeric protein). The precise TLR involved in MyD88 activation was not investigated in this study. However, previous studies have unraveled Ad’s capacity to trigger different TLRs, such as TLR2 (30), TLR4 (17), and TLR9 (14, 30).

Modification of IgG isotype balance in MyD88^−/−^ mice compared to their wild-type counterparts was previously reported for other non-enveloped DNA viruses (31–33) but also for enveloped RNA viruses (34, 35). The role of MyD88 may be linked to its ability to trigger type I IFN production by dendritic cells. This cytokine was shown to promote IgG2b and IgG2c production while reducing IgG1 level (36, 37). MyD88 expressed in B cell could also directly promote isotype switching and affinity maturation as described previously (32, 38).

Several studies have formerly shown that Ad triggers RIG-I pathway (16). Our study revealed that mice deficient in MAVS, a protein acting downstream of RIG-I, did not show modification in total IgG nor specific IgG isotype production against the epitope displayed into the capsid (Figure 6). This suggests that MAVS/RIG-I pathway did not play any significant role in modifying humoral responses either against the epitope or against the transgene product. Alternatively, other innate immune sensor pathways such as TLR/MyD88 may compensate the absence of functional RIG-I/MAVS pathway.

To summarize, our results show for the first time that the efficacy of epitope display strategy depends neither on Ad infection process nor on Ad interaction with its natural receptors. Interestingly, whereas mice deficient in TLR/MyD88 or RIG-I/MAVS pathways mount IgG antibody responses comparable to control mice, we unmasked a key role of TLR/MyD88 pathway in shaping antibody isotype production against the epitope inserted into Ad capsid. Taken as a whole, the present study improved our understanding of molecular bases controlling the efficacy of Ad displaying epitopes on their capsid. The results pave the way for the development of vaccines based on epitope display on Ad capsid.

## Ethics Statement

All animal experiments were approved by Ethics Committee No. 26 in accordance with the European Directive 2016/63 UE and its transposition into French Law.

## Author Contributions

AA performed the experiments, analyzed and interpreted data, and wrote the first draft of the manuscript. NR constructed adenovirus mutants, performed experiments, analyzed data, and corrected the manuscript. PP performed the experiments, analyzed, and interpreted data. LZ performed the experiments and analyzed data. RG and BR provided MAVS and MyD88-deficient mice, respectively. KB supervised the study, designed the experiments, performed the experiments, analyzed data, and wrote the manuscript. All authors read and approved the final version of the manuscript.

## Conflict of Interest Statement

The authors declare that the research was conducted in the absence of any commercial or financial relationships that could be construed as a potential conflict of interest.
